# Age at first birth and risk of urinary incontinence after delivery: a dose–response meta-analysis

**DOI:** 10.1038/s41598-022-19809-x

**Published:** 2022-10-05

**Authors:** Yongcheng Ren, Qing Hu, Haiyin Zou, Meifang Xue, Xinjie Tian, Fuqun Cao, Lei Yang

**Affiliations:** 1 School of Medicine, Institute of Health Data Management, Huanghuai University, Zhumadian, 463000 He’nan People’s Republic of China; 2 Department of Health Examination, Zhumadian Central Hospital, Affiliated Hospital of Huanghuai University, Zhumadian, 463000 He’nan People’s Republic of China; 3 College of Public Health, Zhengzhou University, Zhengzhou, 450001 People’s Republic of China

**Keywords:** Urethra, Risk factors

## Abstract

Studies investigating the impact of age at first birth on urinary incontinence after delivery have reached inconsistent conclusions. We performed this systematic review and meta-analysis of studies assessing the risk of urinary incontinence after delivery, regardless of the type, with age at first birth. MEDLINE via PubMed and Web of science databases were searched up to March 13, 2021. Restricted cubic splines were used to model the dose–response association. Twelve publications were included in this meta-analysis. The summary odds ratio (OR) and 95% confidence interval (CI) per 1-year increase in age at first birth were 1.01 (95% CI (0.99, 1.02)) for urinary incontinence (America: 1.00 (0.99, 1.00); Europe: 1.03 (1.00, 1.06); Asian: 0.99 (0.89, 1.10)). A non-linear dose–response (*P*_nonlinearity_ < 0.01) indicated that age at first birth older than 32 (*P* < 0.05) increases the risk of urinary incontinence. First birth before age 32 make decrease the risk of urinary incontinence after delivery.

## Introduction

Urinary incontinence (UI), is one of the problems during pregnancy and the postnatal period, with a prevalence ranging from 5 to 70%^1,2^. It is not only an organic lesion, but also causes psychological problems such as depression and reduced self-esteem^3,4^, which can seriously affect a woman’s quality of life^5^.

Recent evidence suggests that advanced maternal age at pregnancy, timing of delivery, pregnancy, obstetric trauma and mode of delivery, infant birthweight, infant head circumference, obesity and ageing, and bladder neck hypermobility are the risk factors for UI^6–8^. Age at first birth is associated with mortality^9^, BMI^10^, and cancer^11^. However, studies investigating the effect of age at first birth on UI have reached inconsistent conclusions. A retrospective cohort study shows that younger age at first birth is associated with a higher risk of UI in later life^12^, while another study shows that age at first birth over 25 years is associated with UI^13^. Furthermore, no study has been reported so far on quantitative and comprehensive evaluation of the dose–response association between incidence of UI after delivery and age at first birth. In this study, we performed a dose–response meta-analysis of risk of UI after delivery and age at first birth to provide an evaluation of the existing data. Limited by the number of articles, we did not classify the types of UI after delivery in this meta-analysis. Our aim is to elucidate the shape and strength of the dose–response association between UI after delivery and age at first birth, and to determine the potentially optimal age at first birth for protection against UI after delivery.

## Methods

### Literature search strategy

PubMed and Web of Science databases were searched from their inception until March 13, 2021. The search terms used for the PubMed and Web of science search, including medical subject heading (MeSH) terms and free texts, are provided in Supplemental Table S1. Published studies of age at first birth among women and the incidence or morbidity due to UI after delivery were included if they reported adjusted hazard ratio (HR), relative risk (RR), or odds ratio (OR) estimates and 95% confidence intervals (CIs). The reference lists for all included studies^14–25^ and previous reviews^26–29^ were manually searched for additional relevant studies.

### Study selection

**Figure 1:**
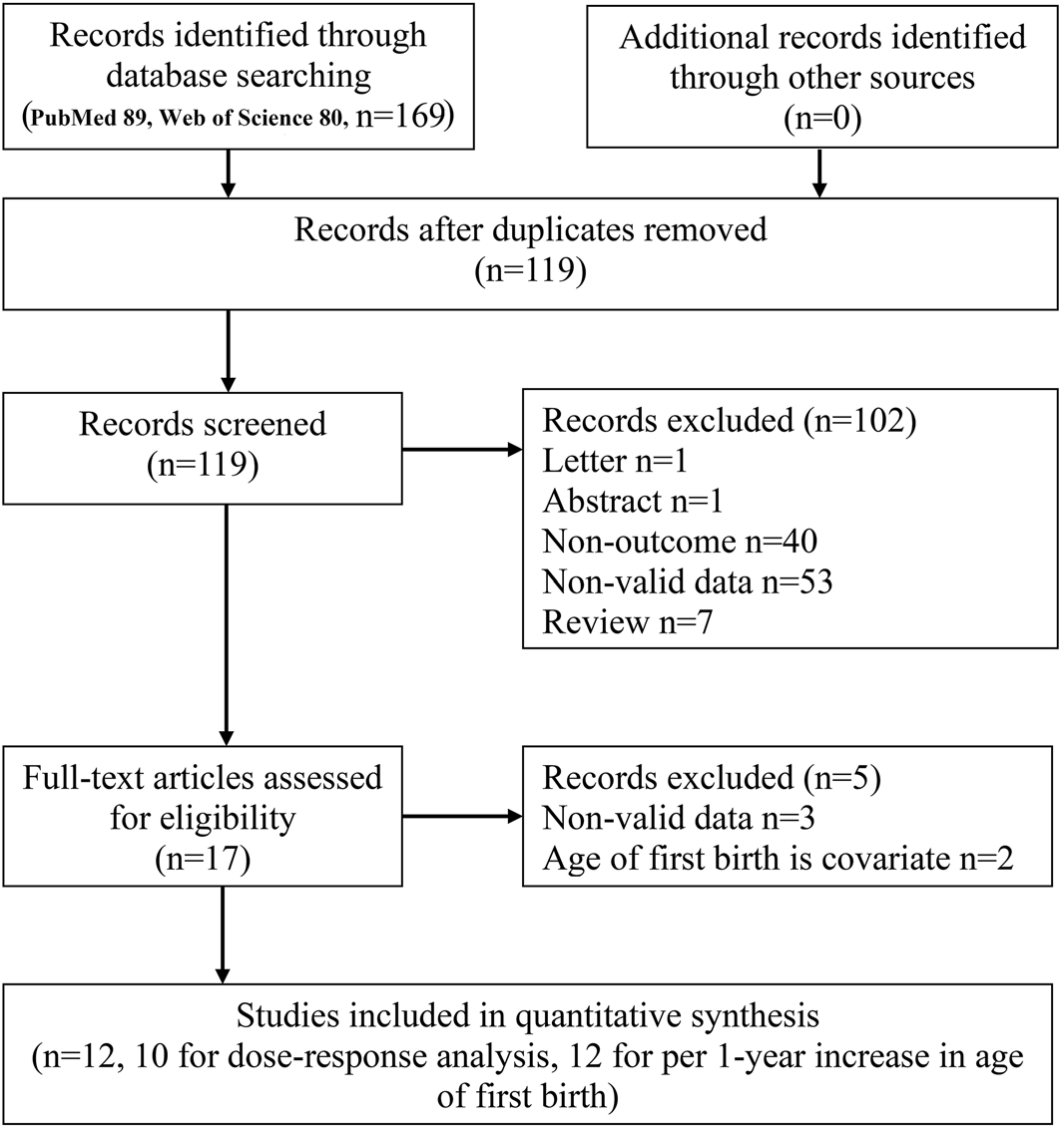
Flow-chart of study selection.

For dose–response analysis, the reports must include a quantitative measurement of at least three categories of age at first birth. Reviews, meta-analyses, duplicate publications, ecological studies and studies without adjusted risk estimates or with unusable data, as well as unpublished studies and grey literature were excluded. When there were duplicate publications for the same study, we chose the publication with the most cases. Literature search and the screening of studies were conducted by RY, ZH, CF, and TX, and RY repeated the screening of the 17 potentially relevant studies identified from the initial screening (Fig. 1). Any discrepancies were resolved in discussion part. The quality of those studies for the following cohort studies was assessed by RY and YL, by using the Newcastle–Ottawa Scale (NOS), which gave a score of 0–9 based on selection, comparability, and outcome assessment^30^ (Supplemental Table S2). RY and YL were also used to assess the quality of those studies for cross-sectional studies, by using Appendix D, Quality Assessment Forms, and Agency for Healthcare Research and Quality, which include 11 items with responses “yes”, “no” and “unclear”^31^ (Supplemental Table S3). We followed the PRISMA criteria to report Meta-analyses of Observational Studies in Epidemiology^32^.

### Data extraction and exposure harmonization

The following data from the studies were extracted into a table (Supplemental Table S4): name of first author, publication year, country or region, sample size, outcome types, category of age at first birth, RRs/HRs/ORs and 95% CIs, and variables adjusted for in the analysis. If the number of cases in each category was missing, these data were inferred based on the total number of cases and the effect size reported. If the exposed person-years or participant numbers were not reported in each category, the groups were assumed to be of equal size^33^, with a lower boundary set to 12^19^, when the lowest category was open-ended.

### Statistical methods

For studies reporting HRs, RR, or ORs for UI after delivery, we assumed the HRs and RRs were approximately equal to ORs^34^. Summary ORs and 95% CIs for UI for each 1-year increase in age at first birth were calculated by using a random effects model^35^, which considered both within- and between-study variation (heterogeneity). A two-tailed *P* < 0.05 was considered statistically significant.

Generalized least-squares regression was used to estimate the study-specific dose–response association^36^. The DerSimonian and Laird random-effects model^35^ was used to pool the study-specific dose–response OR estimates. Study-specific OR estimates were calculated for each 1-year increase in age at first birth and then pooled for a linear association. Potential nonlinear dose–response relationships between age at first birth and UI after delivery were assessed by using restricted cubic splines, with three knots located at the 25th, 50th, and 75th percentiles of the distribution^37^. The *P*-value for nonlinearity was calculated by testing the null hypothesis that the coefficient of the second spline is equal to zero according to a likelihood ratio test^38^.

Heterogeneity was statistically tested by Cochran Q and I^2^^39^. A *P* < 0.10 was considered statistically significant for the Q-statistic. Subgroup analyses were stratified by geographic locations. We performed a sensitivity analysis by excluding one study at a time to assess the stability of the results and potential sources of heterogeneity. Publication bias was evaluated by Begg’s test^40^ and funnel plot examination, and publication bias was indicated at *P* < 0.10. Stata v12.1 (Stata Corp, College Station, TX, USA) was used for analyses.

## Results

**Figure 2:**
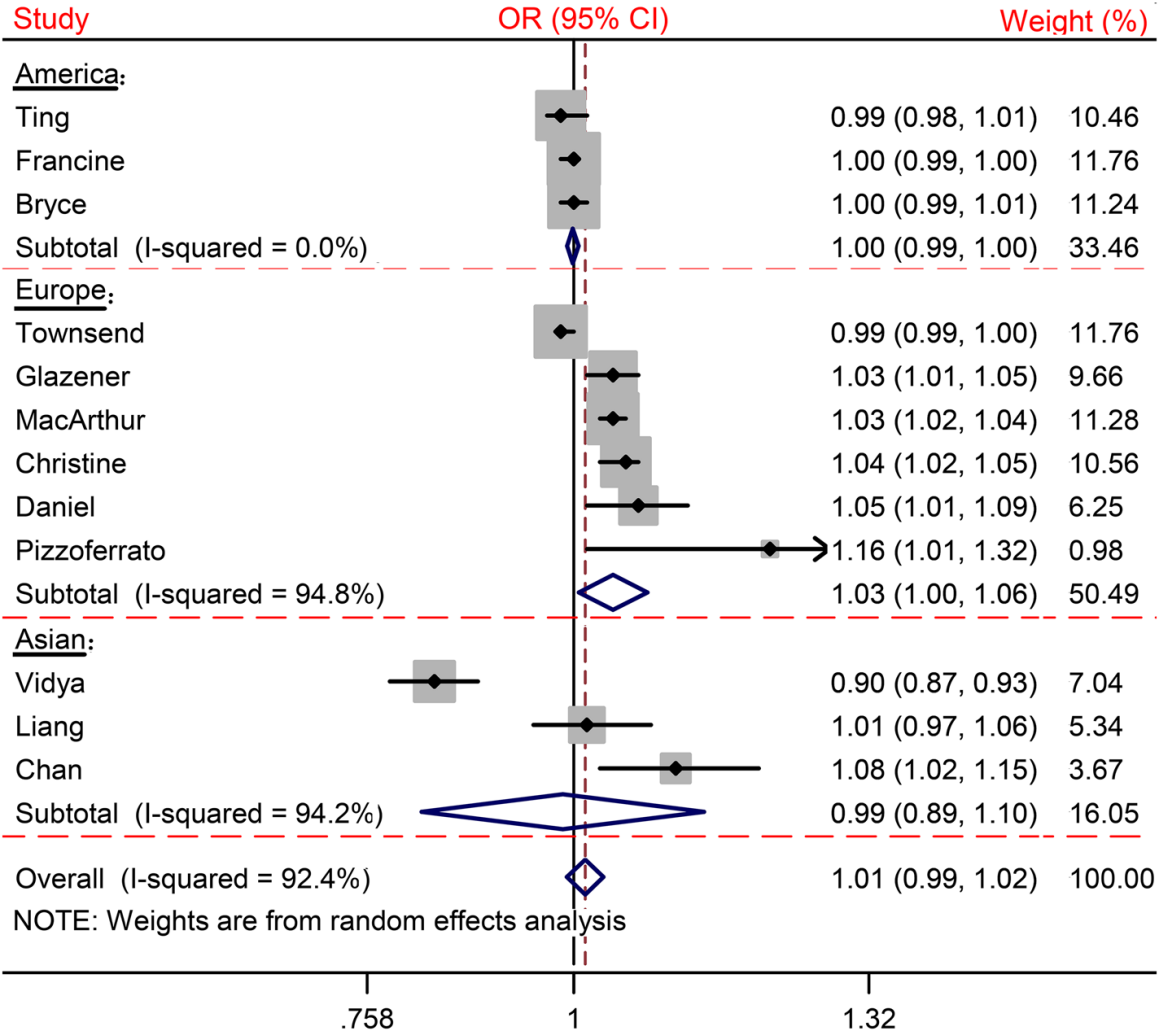
Forest plot of study-specific relative risk statistics for UI per 1-year increment of age at first birth.

**Figure 3:**
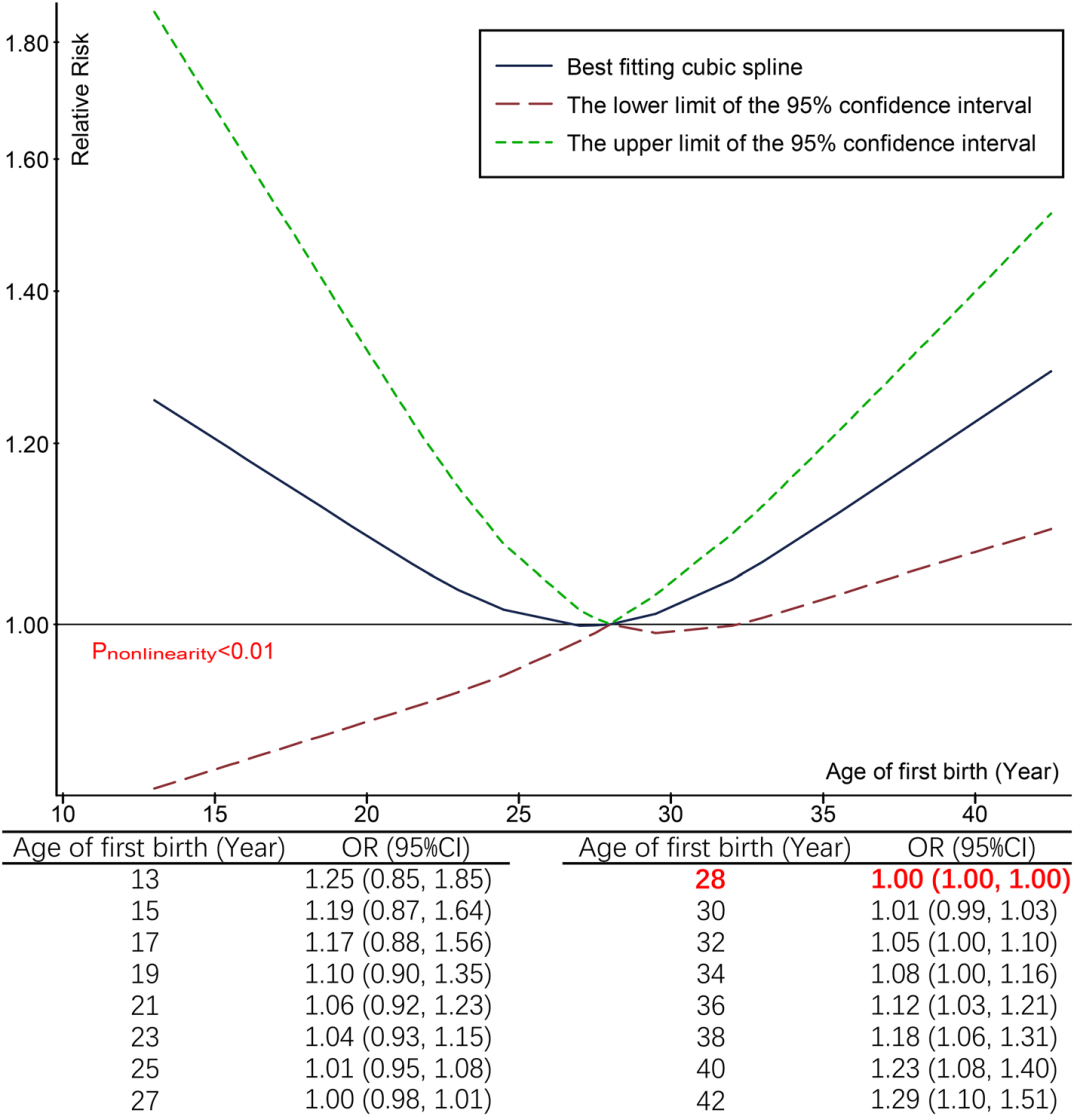
Linear dose–response association between age at first of birth and UI modeled with restricted cubic splines and comparison of the predicted odds ratio point estimates for UI.

There were 169 records identified during our literature search: 119 were evaluated in detail and 17 were considered potentially eligible for inclusion. A total of 12 publications were included in the meta-analysis; Two of these studies reported the risk estimate for UI after delivery of age at first birth on a continuous scale^15,16^. The review included 120,290 participants and all studies involved adult women (≥ 18 years old). Three publications were from America^14,19,23^, six from Europe^15,17,18,20–22^, and three from Asia^16,24,25^. The features of the included studies were summarized in Supplemental Table S4. Figure 1 shows the study selection procedure, Fig. 2 details the analyses for the per 1-year increment of age at first birth, and Fig. 3 details the linear dose–response analyses of the UI after delivery. Supplemental Figure S1 shows the results of publication bias, and Supplemental Fig. S2 details the sensitivity analysis.

### Relative risk for UI after delivery per 1-year increment of age at first birth

A total of twelve publications have been analyzed. The ORs for UI after delivery per 1-year increase of age at first birth ranges from 0.87 to 1.32, and the summary OR is 1.01 (95% CI [0.99, 1.02], *P*_heterogeneity_ < 0.01) (Fig. 2). No publication bias was found (Supplemental Fig. S1, *P* > 0.05). In the sensitivity analysis, the direction and size of the pooled estimates for all results have no change when one single study got removed at a time. (Supplemental Fig. S2). The subgroup analyses found that of all other regions, Europe shows the worst unhealthy effect of a 1-year increment of age at first birth (1.03; [1.00, 1.06]; *P*_heterogeneity_ < 0.01). No unhealthy effect were found in Asia (0.99; [0.89, 1.10]) and America (1.00; [0.99, 1.00]).

### Dose–response association between age at first birth and UI after delivery

Ten publications were included in the nonlinear dose–response analysis after the exclusion of two publications^15,16^ that reported only continuous risk estimates. We identified a positive nonlinear correlation between age at first birth and UI after delivery (*P*_nonlinearity_ < 0.01), showing a U-shaped curve (Fig. 3). Compared to the optimal cut-off value (first birth age = 28 years), UI after delivery risk started at 32 years (1.05; [1.00, 1.10], *P* < 0.05) and significantly increased with the age at first birth (range of age at first birth: 13–42 years) (Fig. 3).

## Discussion

The readiness and soundness of the organism at the time of first birth is primarily linked to its health and survival years and even decades later. Our dose–response analysis found a nonlinear relationship between age at first birth and UI after delivery, with the inflection point at first birth age of 32 years, and the risk increasing with the age at first birth.

Various risk factors such as age, obesity, multiparity, and mode of delivery have been associated with an increased risk of UI^15,41,42^ in women. Numerous epidemiological studies have supported a causal link between vaginal delivery and UI^43^ based on alterations in the nerves, connective tissues and pelvic floor^44,45^. The available evidence suggests that premature birth is associated with a higher reported incidence of heart disease, lung disease, and cancer^46,47^, but the association with UI is inconsistent^14,16,25^. Our meta-analysis provided an updated summary estimates of the association between age at first birth and UI after delivery, and found that increasing menarche age showed a stable and significant association with increased risk of UI after delivery in women. Although this research was not designed to investigate the underlying pathophysiologic mechanisms for the associations revealed, we speculate that the possible reason for this phenomenon was likely that pelvic floor muscles generally are stronger and therefore better for postpartum recovery in women with first birth before 32 years old. In addition, our result did not indicate that age at first birth younger than 28 was beneficial compared to the age at the first delivery which is between 28 and 30 (Fig. 3). According to the evidence that giving a first birth before age 20 is associated with increased mortality^46^, we suggested that the best age range for women to have their first child is 20–32. However, over the last two decades, women have waited to embark on their first pregnancy later and later due to social and economic factors including the expanding role for women in the workforce, with most women having their first child above the age of 35^48,49^. For women who are married and want to have children, interventions on the appropriate age for childbearing are therefore urgent.

Some potential limitations of our meta-analysis should be mentioned. First, we need mean or median exposure of each group to estimate the log ratio of dose response since the data we used were based on quantiles or categories. However, some exposures we used were calculated by inference but not provided by original papers, which have the possibility to cause biased results. Second, due to the limitations of the original reports, there are few studies on urine leakage of different classification. Therefore, we didn’t perform a stratified analysis in the dose–response analysis, and the results of the meta-analysis might be unstable. Third, in the dose–response analysis we could not consider the impact of maternal demographic characteristics and fetal status on urine leakage, and the raw data extracted from the included literature were adjusted for the first child’s weight, parity, and maternal age. Last, we did the data pooling of cross-sectional and cohort studies; sensitivity analysis which has removed one single study at a time found that the direction and size of the pooled estimates for all results remained similar, so we considered the data to be robust. However, further work is still needed to confirm these results due to the small number of literatures we used.

## Conclusion

This meta-analysis used a comprehensive dose–response analysis to elucidate the association between age at first birth and UI after delivery, and confirmed the consistency of our findings through sensitivity analyses. Our results support that older age at first birth (≥ 32 years) increase the risk of UI after delivery. These results may help identify women at increased UI after delivery risk who would benefit from early prevention strategies.

## Supplementary Information

Supplementary Information 1.

Supplementary Information 2.

## Data Availability

All data generated or analyzed during this study are included in this published article [and its Supplementary Information files].
