# Supplementary material for: Age at first birth and risk of urinary incontinence after delivery: a dose–response meta-analysis
Source: Sci Rep. 2022 Oct 5;12:16588. doi: 10.1038/s41598-022-19809-x (PMC9535015; doi:10.1038/s41598-022-19809-x)
Supplement: Supplementary file 2 — Supplementary Information 2. [file 41598_2022_19809_MOESM2_ESM.pdf]

Online Supplement

Age at first birth and risk of urinary incontinence: a dose-response meta-analysis

Running title：age at first birth and urinary incontinence

Supplementary Material

Table S1. Systematic literature review search terms and strategy

**Search terms for PubMed and Web of science**
#1 (Urinary Incontinence [Mesh]) OR (Urination Disorders [Mesh])
#2 ("age at birth of first child" [Title/Abstract]) OR ("age at first reproduction" [Title/Abstract]) OR ("age at first birth" [Title/Abstract]) OR (“first child” [Title/Abstract]) OR (“first reproduction” [Mesh]) OR (“maternal age” [Title/Abstract]) OR (“first birth” [Title/Abstract])
#1 AND #2

Table S2. Quality assessment of included cohort studies

Author (Publication Year)	Newcastle-Ottawa Scale									Total
	Selection				Comparability		Outcome			
	1[a]	2[b]	3[c]	4[d]	5[e]	6[f]	7[g]	8[h]	9[i]	
Francine (2003)	1	1	1	1	1	0	1	1	1	8
Christine (2006)	1	1	1	1	1	1	1	1	1	9
Daniel (2006)	1	1	1	1	1	1	1	1	1	9
Ching (2013)	1	1	1	1	0	1	1	1	1	8
Pizzoferrat (2014)	1	1	1	1	1	0	1	1	1	8
Chan (2014)	1	1	1	1	1	1	1	1	1	9
MacArthur (2016)	1	1	1	1	0	0	1	1	1	7

[a] Representativeness of the exposed cohort; [b] Selection of the non-exposed cohort; [c] Ascertainment of exposure. [d] Demonstration that outcome of interest was not present at start of study. [e] Comparability of cohorts on the basis of the design or analysis (adjusted for age). [f] Comparability of cohorts on the basis of the design or analysis (adjusted for delivery type). [g] Assessment of outcome. [h] Was follow-up long enough for outcomes to occur. [i] Adequacy of follow-up of cohorts.

Table S3. Quality assessment of included cross-sectional studies

Author (Publication Year)	Appendix D. Quality Assessment Forms										
	1	2	3	4	5	6	7	8	9	10	11
Glazener (2006)	yes	yes	yes	yes	no	yes	yes	yes	yes	yes	no
Bowling (2009)	yes	yes	yes	yes	no	yes	yes	yes	yes	yes	no
Vidya (2013)	yes	yes	yes	yes	no	yes	yes	no	yes	yes	no
Townsend (2017)	yes	yes	yes	yes	no	yes	yes	yes	yes	yes	no
Ting (2020)	yes	yes	yes	yes	no	yes	yes	yes	yes	yes	no

1.Define the source of information (survey, record review); 2.List inclusion and exclusion criteria for exposed and unexposed subjects (cases and controls) or refer to previous publications; 3.Indicate time period used for identifying patients; 4.Indicate whether or not subjects were consecutive if not population-based; 5.Indicate if evaluators of subjective components of study were masked to other aspects of the status of the participants; 6.Describe any assessments undertaken for quality assurance purposes (e.g., test/retest of primary outcome measurements); 7.Explain any patient exclusions from analysis; 8.Describe how confounding was assessed and/or controlled; 9.If applicable, explain how missing data were handled in the analysis; 10.Summarize patient response rates and completeness of data collection; 11.Clarify what follow-up, if any, was expected and the percentage of patients for which incomplete data or follow-up was obtained

Table S4. Summary of the characteristics of studies included in this study

ID	Author, published year, country	Study design	Number of total participants	Outcome types	Category of age at first birth (year)	Relative risks (95% confidence intervals)	Adjustment for confounding factors
1	Francine et al., 2003, US	The Nurses' Health Study cohort	83168	Occasional leaking urine	<21	1.12 (0.99, 1.26)	Adjusted for age, race, diabetes mellitus, body mass index, cigarette smoking, stroke, functional limitations, hysterectomy, menopause, and postmenopausal hormone therapy, the number of births.
					21-25	1	
					26-30	1.07 (1.02, 1.12)	
					31-35	1.07 (0.97, 1.18)	
					>35	1.07 (0.89, 1.3)	
				Frequent leaking urine	<21	1.27 (1.13, 1.42)	
					21-25	1	
					26-30	0.99 (0.94, 1.04)	
					31-35	0.99 (0.9, 1.09)	
					>35	1.13 (0.94, 1.36)	
2	Christine et al., 2006, Scotland, England, New Zealand	Longitudinal study	4214	Multiparous	<25	1	mode of first delivery; maternal age at first birth; total number of births at follow up (one/two/three/four or more); and south Asian ethnic origin
					25-29	1.28 (1.07, 1.52)	
					30-34	1.63 (1.32, 2.01)	
					≥35	2.39 (1.72, 3.34)	
				Monotocous	<25	1	
					25-29	1.24 (0.97, 1.57)	
					30-34	1.62 (1.21, 2.16)	

					⩾35	2.36 (1.52, 3.67)	(non-Asian/Asian).
**3**	Glazener et al., 2006, Scotland, England, New Zealand	Questionnaire survey of women	3405	Any	⩽25	1	Delivery type, Gestational age, Birthweight, Head circumference, BMI before pregnancy
					26-29	1.52 (1.18, 1.94)	
					30-34	1.46 (1.1, 1.94)	
					⩾35	2.21 (1.47, 3.33)	
**4**	Daniel et al., 2006, Sweden	Cohort study	229	Stress	Continuous	1.1 (0.9, 1.2)	age and parity, Maternal weight at index delivery, Fetal weight at index delivery, History of perineal rupture, History of instrumental delivery
					19-24	0.9 (0.8, 2)	
					25-30	1	
					31-35	1.4 (0.6, 3.2)	
					36-40	1.7 (0.5, 2.5)	
				Urinary	Continuous	1.1 (0.9, 1.2)	
					19-24	1.2 (0.7, 2.9)	
					25-30	1	
					31-35	1.8 (0.5, 5.6)	
					36-40	1.3 (0.3, 6.8)	
**5**	Bowling et al., 2009, US	Cross-section	5937	Any	⩽16	1.1 (0.8, 1.5)	Maternal race, alcohol and tobacco use, increasing BMI, maternal age, Birth weight, Delivery type
					17-20	0.9 (0.7, 1.2)	
					⩾21	1	
**6**	Ching et al., 2013, China	Cohort study	312	stress	15-24	1	BMI at delivery, BMI at 5 years, Parity, Mode of delivery, Labor duration, Birth weight
					25-34	0.72 (0.28, 1.99)	
					⩾35	1.09 (0.37, 3.29)	
				Urinary	15-24	1	
					25-34	0.72 (0.22, 1.95)	
					⩾35	1.19 (0.33, 4.29)	
**7**	Vidya et al., 2013, India	Cross-section	598	Any	<20	1	
					20-25	0.32 (0.22, 0.46)	
					25-30	0.32 (0.19, 0.54)	
					⩾30	0.46 (0.09, 2.3)	
**8**	Pizzoferrato et al., 2014, French	Cohort study	236	Any	Continuous	0.86 (0.75, 0.98)	BMI at baseline, delta BMI during follow-up, UI during first pregnancy, first child's weight, maternity, age, mode of delivery
**9**	Chan et al., 2014, China	Cohort study	442	Stress	Continuous	1.1 (1, 1.2)	body weight, fetal lie, fetal presentation, engagement of fetal part
						1.05 (0.99, 1.11)	
						1.08 (1.02, 1.15)	

**10**	MacArthur et al., 2016, Scotland, England, New Zealand	Twelve-year longitudinal cohort study	3759	Any	⩽24	1	total number of births, BMI, ethnic origin, onset of labour, perineal trauma, and birth-weight
					25-29	1.38 (1.17, 1.62)	
					30-34	1.78 (1.46, 2.17)	
					⩾35	2.32 (1.68, 3.2)	
**11**	Townsend et al., 2017, Mexican	Cross-section al	15,296	Any	<20	1.18 (1, 1.38)	Age, parity, smoking, age at menopause, postmenopausal HT use, BMI at age 18, current BMI, waist-to-hipratio, asthma, high blood pressure, diabetes, and neurological disease
					20-24	1	
					25-29	0.93 (0.82, 1.05)	
					⩾30	0.98 (0.84, 1.15)	
**12**	Ting et al., 2020, Rio Grande	Cross-section al	2694	Any	12-19	1.36 (1.04, 1.76)	Maternal age, Maternal skin color, Maternal schooling, Weight at the end of pregnancy, Living with partner, Delivery type
					20-29	1	
					⩾30	1.59 (1.01, 2.51)	

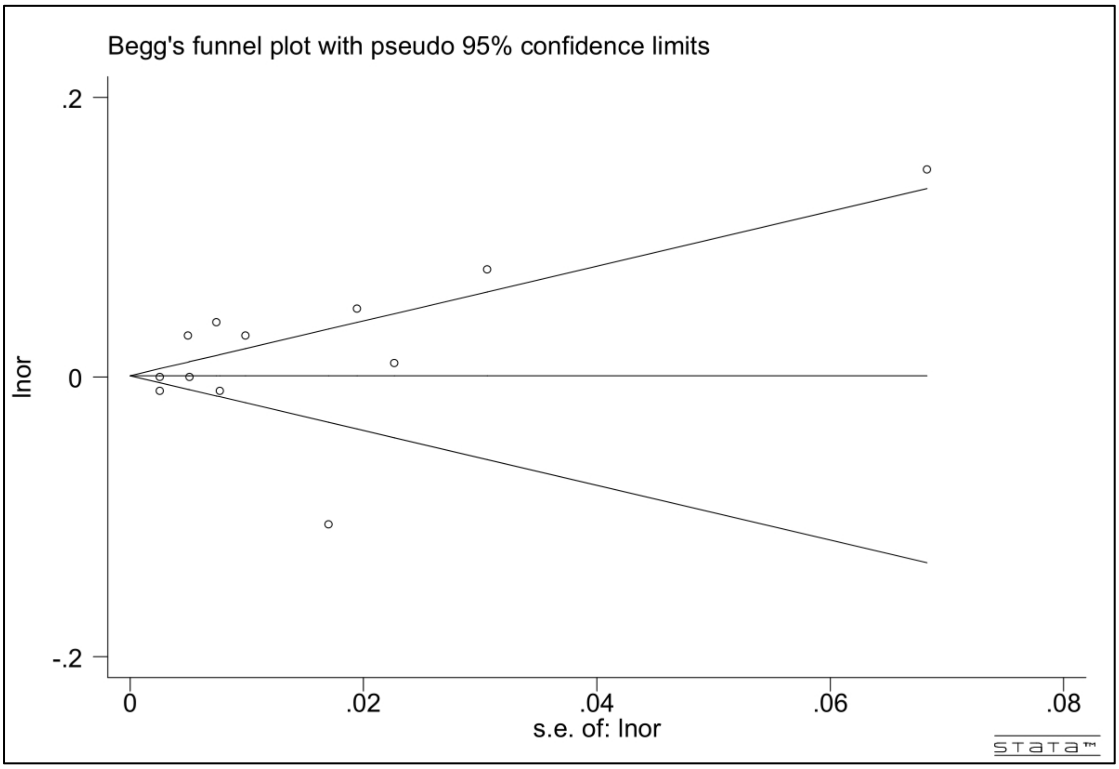

Figure S1. Funnel plot for publication bias for risk of UI with age at first birth

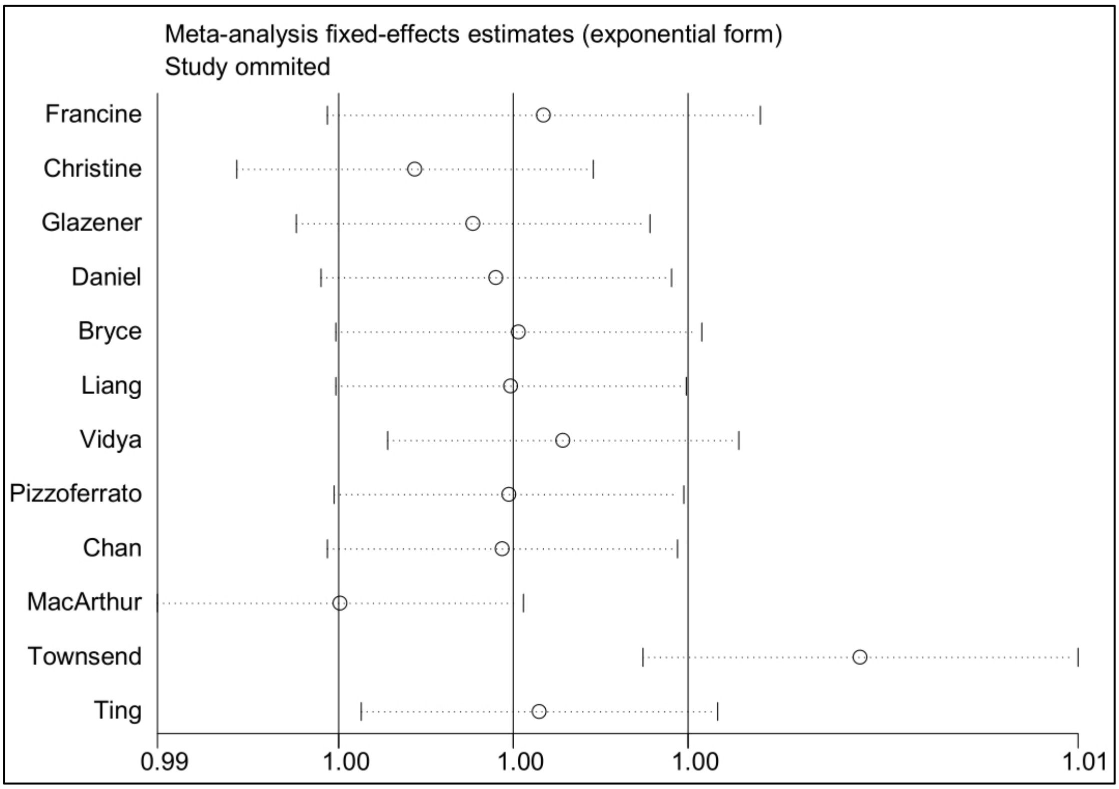

Figure S2. Funnel plot for sensitivity analysis for risk of UI with age at first birth
